# mRNA expression profiling and pathway analysis of chronic intermittent hypoxia–induced pancreatic injury in ob/ob mice

**DOI:** 10.3389/fphys.2026.1740223

**Published:** 2026-02-25

**Authors:** Yaopeng Guo, Shengquan Huang, Jiayi Lin, Chaowei Li, Ruhai Lin, Qingshi Chen

**Affiliations:** 1 Department of Endocrinology and Metabolism, The Second Affiliated Hospital of Fujian Medical University, Quanzhou, China; 2 Department of Pathology, The Second Affiliated Hospital of Fujian Medical University, Quanzhou, China; 3 Department of Gastroenterology, The Second Affiliated Hospital of Fujian Medical University, Quanzhou, China

**Keywords:** chronic intermittent hypoxia, ob/ob mice, obstructive sleep apnea, pancreatic injury, protein–protein interaction network, RNA sequencing

## Abstract

**Background:**

Increasing evidence suggests that messenger RNA (mRNA) is centrally involved in the initiation and progression of various diseases. However, its involvement in pancreatic injury resulting from obstructive sleep apnea (OSA) continues to be incompletely elucidated. The present investigation aimed to characterize mRNA expression changes using a murine model of chronic intermittent hypoxia (CIH) to provide new insights into the mechanisms underlying OSA-associated pancreatic injury.

**Methods:**

An ob/ob murine model for pancreatic injury triggered by CIH was established. RNA sequencing (RNA-seq) was conducted to detect differentially expressed mRNAs, and subsequently Gene Ontology (GO) and Kyoto Encyclopedia of Genes and Genomes (KEGG) pathway analyses were applied to delineate the associated functional annotations and signaling cascades. Furthermore, several selected mRNAs were validated using reverse transcription PCR (RT-qPCR). Finally, we constructed a protein–protein interaction (PPI) network to delineate the interplay among the protein targets of the differentially expressed genes (DEGs).

**Results:**

In a mouse model of CIH-induced pancreatic dysfunction, 481 mRNAs were upregulated and 165 were downregulated. KEGG enrichment analysis indicated that the NOD-like receptor signaling pathway is implicated in CIH-induced pancreatic dysfunction. Subsequently, several differentially expressed mRNAs were subjected to RT-qPCR validation. On the basis of these data, a subset of DEGs were selected to construct a PPI network.

**Conclusion:**

Overall, we identified 646 DEGs in the CIH mouse model. These results may offer important perspectives on the pathophysiological processes that underlie OSA–induced diabetes mellitus.

## 1 Introduction

Obstructive sleep apnea (OSA) is a prevalent sleep-disordered breathing disorder marked by repeated upper-airway collapse during sleep, which results in chronic intermittent hypoxia (CIH) along with sleep fragmentation (Tasali et al., 2025). Relevant investigations have documented that obstructive sleep apnea is linked to a wide spectrum of cardiovascular diseases (Li et al., 2025). OSA is also associated with hepatic dysfunction, large clinical studies report a higher prevalence of nonalcoholic fatty liver disease in OSA patients (Tang et al., 2023), likely via intermittent hypoxia–induced oxidative stress and inflammatory responses (Wang et al., 2024). At the level of the central nervous system, OSA has been associated with neurocognitive impairment and memory deficits (Miyo et al., 2025). Importantly, emerging clinical and animal data indicate that chronic intermittent hypoxia in OSA can directly injure the pancreas: human studies report OSA-associated pancreatic β-cell dysfunction and insulin resistance (Umano et al., 2023; Yin et al., 2025) and murine CIH models demonstrate oxidative stress, inflammatory signaling and apoptosis of pancreatic islet cells (Zeng et al., 2024). To date, the molecular mechanisms underlying OSA-related pancreatic injury remain poorly understood.

Messenger RNA (mRNA) is a single-stranded RNA transcript synthesized from DNA that carries the codon sequence needed for translating proteins at ribosomes, acting as the central intermediary in gene expression and protein synthesis in cells (Hou et al., 2024). mRNA expression profiling through high-throughput methods (such as RNA-seq) has become a routine and powerful approach for mapping global gene-expression changes across diseases, revealing dysregulated pathways and pinpointing therapeutic targets (Yang et al., 2020). For instance, in cardiovascular research, RNA-seq of peripheral-blood–derived mononuclear cells from coronary artery disease (CAD) patients uncovered hundreds of differentially expressed genes; notably, the genes BTRC and UBE2D2 were validated as candidate blood biomarkers for CAD diagnosis, highlighting new therapeutic possibilities (Chen et al., 2021). In inflammation research, bulk RNA-seq of aortic aneurysm tissue distinguished inflammatory from non-inflammatory cases by revealing immune-related signature genes and candidate drug targets for aortitis (Hur et al., 2022). Collectively, these examples illustrate that mRNA expression profiling elucidates disease mechanisms and highlights intervention points. Building on this paradigm, it is logical to apply transcriptomics to pancreatic injury under CIH. CIH triggers pancreatic inflammation, pathological lesions, and β-cell apoptosis through the activation of relevant signaling pathways (Wang et al., 2020; Uchiyama et al., 2021). Therefore, profiling the pancreatic transcriptome in CIH models should reveal the hypoxia-driven gene-expression changes underlying injury and suggest novel targets for intervention. Such an approach is expected to shed light on the molecular basis of CIH-induced pancreatic injury and guide the identification of new therapeutic targets. However, few studies have characterized the alterations in mRNA expression within the pancreas following CIH-induced injury.

In this study, pancreatic tissues from mice exposed to CIH were examined by hematoxylin-eosin (HE) staining. Subsequently, transcriptome profiling by mRNA sequencing was performed to identify differentially expressed genes. Selected differentially expressed mRNAs underwent validation by real-time PCR (RT-qPCR). Gene Ontology (GO) and Kyoto Encyclopedia of Genes and Genomes (KEGG) analyses were conducted to identify associated biological processes and pathways. Finally, protein–protein interaction (PPI) networks were assembled from the STRING repository. In conclusion, our study is the first to delineate the altered mRNA expression profile and potential functions in the pancreas of CIH-injured ob/ob mice, which may provide novel therapeutic targets for the clinical management of OSA-induced diabetes mellitus.

## 2 Materials and methods

### 2.1 Animal

In the present work, we employed male ob/ob mice obtained from Shanghai SLAC Laboratory Animal Co., Ltd. Mice were randomized into Control and CIH groups (n = 3/group) and maintained under identical conditions except for the daily 8-h CIH exposure. All outcome assessments were performed in a blinded manner. No animals or samples were excluded during the experimental procedures or subsequent analyses. All experimental procedures complied with the U.S. National Research Council’s Life Sciences Committee, Laboratory Animal Resources Institute, Guidelines for the Care and Use of Laboratory Animals. The Institutional Ethics Committee at the Second Affiliated Hospital, Fujian Medical University, granted approval for this research study, and all mice can drink water and eat freely.

### 2.2 CIH protocol

The CIH protocol was implemented as previously described (Li et al., 2024a) and is consistent with well-established rodent models of OSA that aim to mimic the chronic intermittent hypoxia characteristic of moderate to severe sleep apnea (Barnes et al., 2022). This regimen, involving rapid cycles of hypoxia and reoxygenation, has been widely used to induce OSA-associated metabolic and pancreatic dysfunction. In short, the mice allocated to the CIH group were housed within purpose-built hypoxic chambers. This chamber is coupled with the gas-control delivery system that enables a pure-nitrogen stream to lower O_2_ down to 6% in 60 s. Subsequently, the control system further enables rapid oxygen replenishment, thereby rapidly reoxygenating to 21% O_2_ in 60 s. Each 2-min CIH bout repeats at a frequency of 30 cycles per hour over 8 h daily, lasting for an overall duration of 8 weeks. The chamber was equipped with integrated sensors that continuously monitored intra-chamber O_2_ concentration, CO_2_ concentration, temperature, and humidity in real time, with readouts displayed on a visualized control interface to allow ongoing observation and timely adjustments to maintain the programmed oxygen profile and stable environmental conditions. In the control cohort, indoor air was continuously supplied to the room throughout the experimental period. After 8 weeks of CIH exposure, anesthesia was induced with inhaled isoflurane (3%–4%) and maintained at 1.5%–2.0%. Under a deep plane of anesthesia, all mice were euthanized by cervical dislocation performed by trained personnel. Subsequently, pancreatic tissues were carefully isolated on ice to preserve tissue integrity. All procedures complied with the AVMA Guidelines.

### 2.3 HE staining

Pancreatic tissues were harvested and rinsed in 0.9% saline, immersion-fixed with 4% formaldehyde, dehydrated and paraffin-embedded. Five-micrometer sections were cut and subjected to HE staining. Morphological changes were subsequently evaluated under a light microscope.

### 2.4 RNA extraction and RNA-Sequencing

Total RNA was isolated from mouse pancreatic tissues using the MJzol Animal RNA Isolation Kit in accordance with the manufacturer’s protocol. RNA was purified with the RNAClean XP Kit and treated with the RNase-Free DNase Set. RNA integrity was evaluated using an Agilent 2,100 Bioanalyzer and an Agilent 4,200 TapeStation. Purified total RNA was used to construct mRNA sequencing libraries; library fragment size distribution was evaluated with the Agilent 4,200 TapeStation. Sequencing was conducted using an Illumina NovaSeq 6,000 platform following standard procedures. Raw sequencing reads were preprocessed to remove adapter contamination and low-quality bases; reads containing excessive ambiguous nucleotides were discarded, and overly short reads were filtered out. rRNA-derived reads were removed by alignment to rRNA reference sequences. The quality of the cleaned data was assessed using FastQC. Clean reads were aligned to the *Mus musculus* reference genome GRCm39 (Ensembl release 111) using HISAT2, and alignment statistics were summarized with standard alignment-processing tools. Transcript abundance was quantified using StringTie.

### 2.5 Differential expression analysis

Raw sequencing reads were processed and aligned to the reference genome, and gene-level counts were obtained. Differential expression analysis between the control and CIH groups was performed using DESeq2. Multiple testing was corrected using the False Discovery Rate (FDR) method. Genes with |log2FC|≥1 and a q-value ≤0.05 were identified as differentially expressed. Volcano plots and heatmaps were generated to visualize the expression patterns of differentially expressed genes (DEGs). These results provided the basis for subsequent functional enrichment analyses.

### 2.6 Functional enrichment analysis

GO enrichment and interrogation of KEGG pathways were performed to investigate the higher-order roles of the differentially expressed mRNAs. GO analysis stratified genes across hierarchical tiers and uncovered regulatory gene networks inferred from biological processes and molecular functions. KEGG pathway analysis provided insights into signaling pathways and disease-related processes, thereby offering a basis for functional characterization of the genes. GO and KEGG enrichment analyses were performed using annotated genes in each database as the background universe, with enrichment significance corrected for multiple testing. P values below 0.05 were deemed statistically significant.

### 2.7 RT-qPCR

Pancreatic tissues from ob/ob mice were lysed and homogenized in TRIzol reagent. Total RNA was subsequently isolated from the homogenate in line with the reagent manufacturer’s instructions. One microgram of total RNA underwent reverse transcription to generate complementary DNA (cDNA) using a commercial first-strand cDNA synthesis kit under standard conditions. RT-qPCR was performed using 2 × ChamQ Blue Universal SYBR qPCR Master Mix on an Applied Biosystems ViiA 7 Real-Time PCR System. Thermal cycling was programmed as an initial denaturation at 95 °C for 5 min, followed by 40 cycles consisting of denaturation at 95 °C for 10 s and annealing/extension at 60 °C for 30 s. Each reaction was run in triplicate, and a melting curve analysis was conducted at the end of PCR to confirm amplification specificity. Relative expression was derived via the 2^−ΔΔCT^ method, normalizing to GAPDH. The primer sequences of the analyzed genes are listed in Table 1.

**TABLE 1:** Primer sequences.

Primer	Sequence (5′-3′)	Length (bp)
Reg3b	F:GGAGGTGGATGGGAATGGAG R:ACAAGCTGCCACAGAAAGCA	100
Reg3g	F:CCGACACTGGGCTATGAACC R:CCACAGTGATTGCCTGAGGA	113
Reg3a	F:GCTCTCCTGCCTGTTGTTTG R:TAAGGCATAGCAGTGGGAGC	121
Gnai1	F:AGCAGTACAAGGCAGTGGTC R:CACGAAAAGTTGGCGAGCAT	128
KIF9	F:GTGACCAAGGAACACGGTGA R:GGGTCTGGATGGGTCGGTAT	109
Gm56125	F:CCACAACCAGCGCAGACT R:GCCTCCTAGTCGTGCTTGAG	86
GAPDH	F:ATGTGTCCGTCGTGGATCTG R:AAGTCGCAGGAGACAACCTG	142

### 2.8 PPI network analysis

DEGs were mapped to their corresponding protein products and queried in the STRING database to retrieve experimentally supported and curated protein–protein associations (interaction confidence score = 0.4) (Szklarczyk et al., 2023). For PPI construction, the top 50 upregulated and top 50 downregulated DEGs ranked by |log2FC| were selected. The resulting network was imported into Cytoscape, and densely connected modules were identified with the MCODE plugin (Degree cutoff = 2, Node score cutoff = 0.2, K-core = 2) to highlight putative protein complexes and functional clusters.

### 2.9 Statistical analysis

Statistical comparative analyses of mRNA expression levels across the two groups were conducted with Student’s t-test. The data obtained from independent experiments and statistical analyses were expressed as the mean ± standard deviation (SD) using SPSS (version 27.0.1) and Prism 10.0 (GraphPad). The threshold for statistical significance was set at P < 0.05.

## 3 Results

### 3.1 CIH-induced pancreatic injury

**FIGURE 1:**
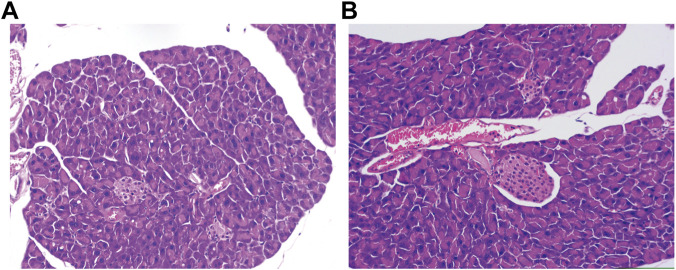
Impact of CIH on the pancreas. **(A)** Histologic images of the pancreas from normoxic control mice demonstrating intact parenchymal organization. **(B)** Histologic images of the pancreas from CIH-exposed mice demonstrating structural abnormalities.

Representative HE staining revealed clear morphological differences between groups. As shown in Figure 1, in controls, pancreatic architecture was preserved, with orderly acinar lobules, intact acinar cell polarity, narrow interstitial spaces, and well-circumscribed islets. In contrast, CIH-exposed pancreata displayed conspicuous structural disarray characterized by acinar cell swelling and vacuolization, cytoplasmic granule depletion, widened interlobular spaces consistent with interstitial edema, and focal inflammatory cell infiltration.

### 3.2 Different expression profiles of mRNAs

**FIGURE 2:**
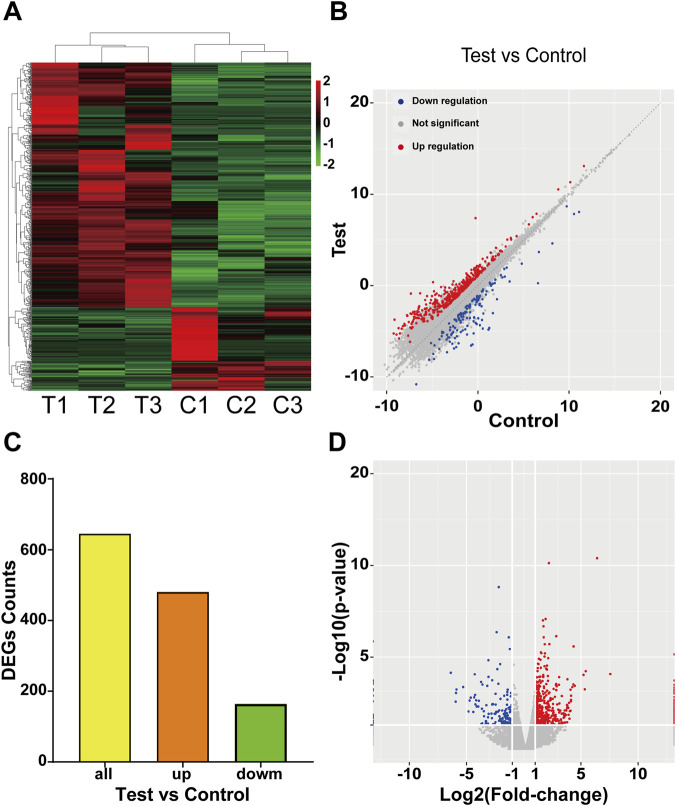
Differentially expressed mRNAs in pancreatic tissue of CIH-exposed mice. **(A)** Hierarchical clustering of DEGs. **(B)** Scatter plot of differential mRNA expression across the CIH and control groups; red points denote mRNAs with increased expression, and blue points denote mRNAs with decreased expression. **(C)** Bar chart comparing the numbers of upregulated and downregulated mRNAs between the hypoxia and control groups. **(D)** Volcano plot highlighting significant differences in mRNA expression across the CIH and control groups; red points mark mRNAs with increased expression, and blue points mark mRNAs with decreased expression. C, control group; T, chronic intermittent hypoxia group.

Sequencing analysis revealed 646 DEGs between CIH and control pancreatic tissues. Hierarchical clustering (Figure 2A) demonstrated divergent mRNA expression profiles across specimens. The scatter and bar plots effectively illustrated the expression disparities between the CIH and control cohorts (Figures 2B,C). The scatter plot indicated that the majority of mRNAs exhibited less than a one-fold change across the two groups (black dots). The volcano plot (Figure 2D) displayed a clear distribution of differential expression, with 481 mRNAs showing significant upregulation (in red) and 165 exhibiting downregulation (in blue).

### 3.3 Validation of DEGs using RT-qPCR

**FIGURE 3:**
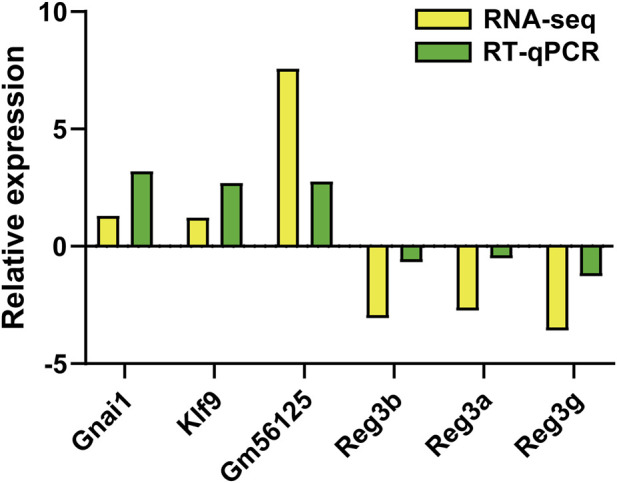
Validation of dysregulated mRNAs. The relative expression levels of six candidate mRNAs were quantified by RT-qPCR. Upward direction indicates gene upregulation, whereas downward direction indicates gene downregulation.

To validate the mRNA sequencing results, six differentially expressed genes were randomly selected for verification by RT-qPCR. As shown in Figure 3, RT-qPCR results showed that three genes (Gnai1, KIF9, Gm56125) were upregulated and three genes (Reg3b, Reg3a, Reg3 g) were downregulated. These results confirmed the consistency between the RNA-seq data and the RT-qPCR findings, thereby validating the reliability of the RNA-seq results.

### 3.4 GO and KEGG analyses

**FIGURE 4:**
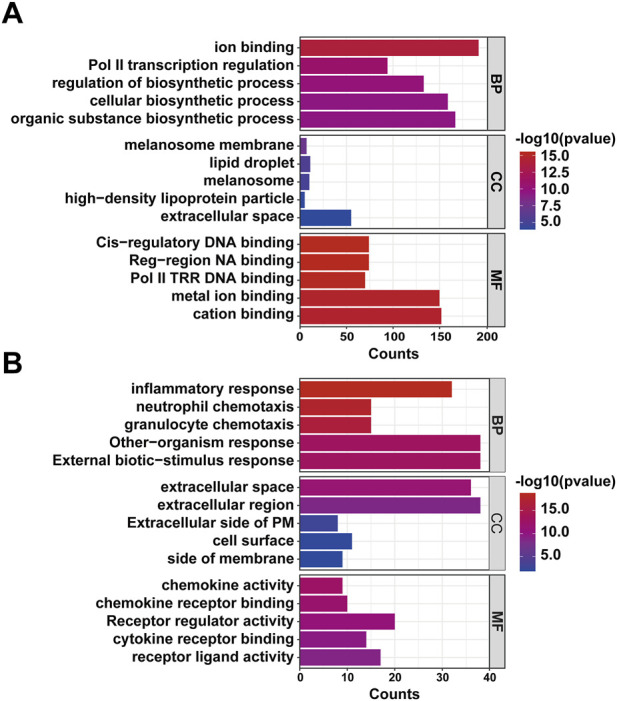
GO enrichment analysis for genes with differential expression. **(A)** Five most enriched GO annotations for upregulated mRNAs; **(B)** Five most enriched GO annotations for downregulated mRNAs.

**FIGURE 5:**
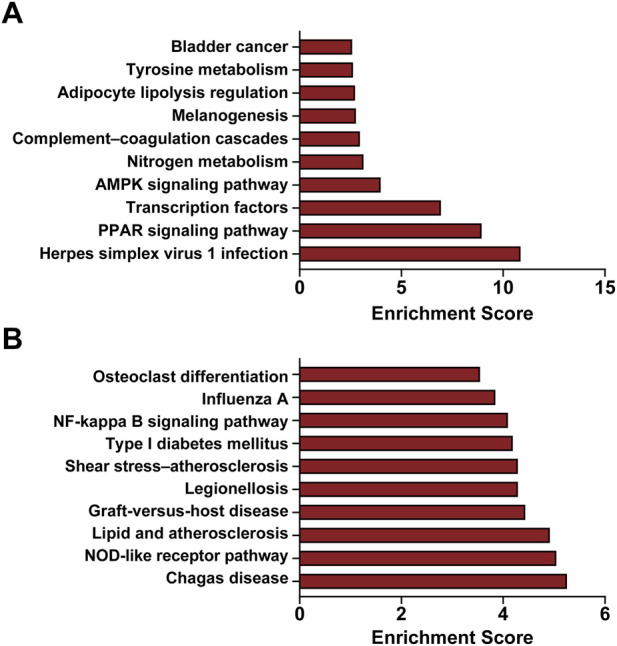
KEGG pathway enrichment analysis of differentially expressed mRNAs. **(A)** Enriched KEGG pathways associated with upregulated mRNAs; **(B)** Enriched KEGG pathways associated with downregulated mRNAs.

To further delineate the functional implications of the DEGs, we performed GO and KEGG enrichment analyses to clarify their potential roles in the pathogenesis of CIH-induced pancreatic dysfunction in ob/ob mice. As shown in Figures 4A,B, the DEGs were significantly over-represented across GO domains—Biological Process (BP), Cellular Component (CC), and Molecular Function (MF). In BP, prominent terms included cellular biosynthetic process; in CC, genes were enriched in the extracellular region; and in MF, terms such as metal ion binding were highlighted. Collectively, these findings indicate that the identified DEGs likely contribute specifically to pancreatic injury under CIH conditions in ob/ob mice. KEGG analysis further demonstrated that these genes mapped predominantly to pathways linked to immune and inflammatory responses, metabolic and energy regulation, and lipid metabolism–related disorders. Notably, most upregulated DEGs were associated with the Herpes simplex virus 1 infection pathway, whereas downregulated DEGs were chiefly enriched in the Chagas disease pathway (Figures 5A,B). To complement DEG-based enrichment, rank-based GSEA was performed using the full ranked gene list, and representative enrichment plots are provided in the Supplementary Material (Supplementary Figures S1,S2).

### 3.5 PPI network

**FIGURE 6:**
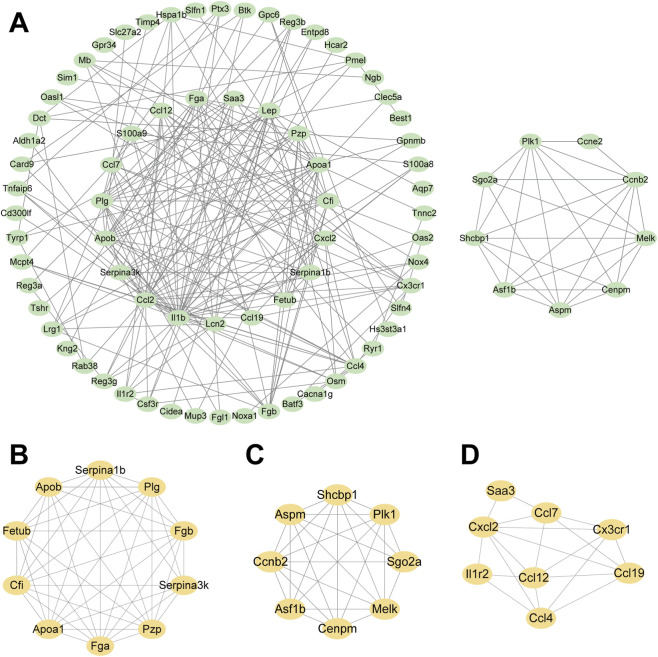
PPI network construction. **(A)** Protein–protein interaction network derived from DEGs. **(B–D)** Top three high-scoring protein interaction modules identified by the MCODE algorithm in C.ytoscape.

The selected DEGs were input into STRING to build a PPI network. The derived network was graphically displayed in Cytoscape (Figure 6A). Subsequent module detection within the PPI network was conducted in Cytoscape using the MCODE plugin. The top three interaction modules (highest scoring clusters) were extracted (Figures 6B–D). The key protein interactions within these highly connected modules may represent potential therapeutic targets for CIH-induced pancreatic injury.

## 4 Discussion

In the study, we performed the first comprehensive analysis of the pancreatic mRNA expression profile in ob/ob mice subjected to CIH. This transcriptomic approach revealed extensive changes in gene expression across key pathways related to insulin secretion, inflammation, and metabolic regulation. These molecular alterations suggest that, in the context of obesity and diabetes, hypoxic stress exacerbates pancreatic dysfunction and may accelerate the progression of type 2 diabetes. By addressing a previously unexplored aspect of hypoxia-induced pancreatic injury, our study fills a critical gap in understanding how CIH contributes to diabetes pathogenesis. This highlights the treatment potential of mRNA in the clinical handling and care of these conditions and offers an encouraging avenue for forthcoming investigation and therapeutic innovation. These results deepen our insight into the mechanistic contribution of altered mRNA expression to the pathogenesis of OSA-related diabetes.

It is well established that OSA can induce systemic complications across multiple organ systems. For instance, this sleep disorder contributes to cardiovascular pathologies (Javaheri et al., 2024), promotes hepatic injury characterized by nonalcoholic fatty liver disease (Hany et al., 2023), and is strongly linked with metabolic syndrome and related metabolic derangements (Giampá et al., 2023). In particular, the chronic intermittent hypoxia characteristic of OSA is now recognized to adversely affect the pancreas via multiple mechanisms (Mokhlesi et al., 2021). For example, repeated hypoxic episodes can induce peripheral insulin resistance, impair pancreatic β-cell function, and provoke chronic low-grade inflammation, ultimately compromising pancreatic integrity (Lv et al., 2023). Accordingly, OSA has been identified as a standalone risk determinant for glucose intolerance and type 2 diabetes mellitus (Gentile et al., 2025), underlining the connection between sleep-disordered breathing and pancreatic metabolic dysfunction.

Messenger RNA is the single-stranded RNA intermediary that carries genetic instructions from DNA to the protein synthesis machinery (Qin et al., 2022; Lu et al., 2024). Given this central role, it is not surprising that dysregulation of mRNA expression has been implicated across a broad spectrum of diseases, and such transcriptomic aberrations can critically influence disease progression and treatment responses (Stevens et al., 2021). These mRNA expression changes are not merely correlative but often functionally significant, reflecting underlying molecular mechanisms of pathology and revealing potential points of intervention. Accordingly, mRNA expression profiles have emerged as valuable biomarkers for disease diagnosis and prognosis (Pham et al., 2024). For example, transcriptome-based assays can distinguish cancer subtypes and guide clinical decision-making based on gene signature patterns (Saju et al., 2025). Likewise, mRNA itself is gaining recognition as a therapeutic target and modality: pathogenic transcripts can be modulated or silenced via antisense oligonucleotides or RNA interference, and exogenous mRNA can be delivered as novel treatments (Qin et al., 2022). These advances underscore the translational significance of mRNA profiling in modern medicine, where transcriptomic data are increasingly leveraged in precision diagnostics and in drug development to identify new molecular targets (Yang et al., 2020). However, few studies have directly linked mRNA expression with CIH-induced pancreatic dysfunction. Therefore, we employed leptin-deficient ob/ob mice to elucidate the role of mRNA in the pathogenic process of pancreatic impairment triggered by CIH.

This study employed mRNA sequencing to characterize differential mRNA expression during CIH-induced pancreatic injury in mice. We observed marked transcriptomic differences between the CIH and control groups, characterized by 481 mRNAs upregulated and 165 downregulated. The categories of these DEGs were further delineated. In addition, six representative mRNAs were arbitrarily chosen for RT-qPCR verification, and the expression trends aligned with the sequencing results, supporting the robustness of our findings and highlighting the contribution of mRNA changes to the initiation and progression of OSA-associated pancreatic damage. The rapid advances in mRNA–disease association research may, in turn, inform the formulation of diagnostic and therapeutic approaches for diabetes attributable to OSA.

We performed GO and KEGG enrichment analyses of these DEGs to further delineate their biological functions in CIH-induced pancreatic dysfunction. GO and KEGG pathway enrichment analyses reveal significant insights into the molecular mechanisms of OSA-related pancreatic damage. Upregulated GO terms such as ion binding and regulation of biosynthetic processes suggest enhanced metabolic and regulatory activities, while enrichment of lipid droplet components points to lipid metabolism alterations, consistent with findings in metabolic disorders (Zadoorian et al., 2023; Hilton et al., 2023). Downregulated inflammatory response and chemokine activity terms reflect suppressed immune signaling, aligning with reports of immune dysregulation in pancreatic diseases (Ju et al., 2024). KEGG pathway analysis indicates activation of the PPAR signaling pathway, a crucial regulator of lipid metabolism and inflammation, implicated in diabetes and pancreatitis. Conversely, downregulated pathways like the NOD-like receptor signaling pathway, involved in innate immunity and inflammation, have been associated with chronic inflammatory processes in pancreatic injury (Liu et al., 2023). These findings collectively underscore the balance between metabolic regulation and immune response in OSA-related pancreatic damage, corroborated by recent studies highlighting these pathways’ roles in related conditions.

Notably, our enrichment analysis identified several KEGG terms associated with infectious or systemic diseases, such as “Herpes simplex virus 1 infection” and “Chagas disease”. In our research, the enrichment of these pathways was primarily driven by the differential expression of core innate immune mediators, specifically IL-1β, the Ccl2 chemokine axis (including Ccl3 and Ccl12), and the interferon-stimulated gene Oas2. IL-1β serves as a critical effector of inflammasome-mediated sterile inflammation. Given that CIH is known to amplify inflammasome/IL-1β-centric inflammatory cascades, this signaling is likely intrinsically linked to islet immune homeostasis and β-cell functional states (Li et al., 2024b). Concurrently, acting as a canonical driver of monocyte/macrophage trafficking, the Ccl2–Ccr2 axis may exacerbate local cytokine signaling and tissue remodeling, providing a mechanistic link between CIH-induced chemokine induction and pancreatic immune alteration (Riddy et al., 2021). Furthermore, Oas2 functions as a dsRNA-sensing, interferon-inducible antiviral factor within the OAS–RNase L network. Its upregulation suggests that CIH may also perturb nucleic acid sensing and interferon-related defense programs, which are frequently categorized under “viral infection” annotations in KEGG (Yang et al., 2024). Collectively, these findings support the interpretation that the appearance of “infection” pathway terms primarily reflects a profound remodeling of host innate immunity in the pancreas under CIH stress, rather than an infectious etiology.

Building on the GO/KEGG results, we constructed a PPI network to contextualize the DEGs at the systems level. Network topology highlighted densely connected modules enriched for lipid metabolism and innate immune signaling, consistent with PPAR and NOD-like receptor pathways. Centrality analysis pinpointed hub proteins that may integrate metabolic reprogramming with inflammatory control under CIH. These hubs and modules provide mechanistic anchors beyond single-gene effects and nominate tractable candidates for functional validation. Prioritizing these nodes may clarify causal axes linking dyslipidemia, immune modulation, and pancreatic injury in OSA. However, our study did not delve into the complex mechanisms underlying the identified DEGs, leaving a substantial gap in understanding their functions. Future investigations should elucidate the biological functions and signaling pathways associated with these mRNAs to clarify their roles in CIH-induced pancreatic injury.

Although we systematically characterized mRNA expression patterns in ob/ob mice subjected to CIH-induced pancreatic injury, our study nonetheless has several limitations. First, the key mRNAs identified in this study lack functional validation. Although our transcriptomic and bioinformatic analyses revealed potential pathways and hub genes, these findings remain hypothesis-generating and primarily serve as a foundation for subsequent research. Future mechanistic experiments will be conducted to determine the causal relationships between the identified transcripts and pancreatic injury. Second, only a subset of the differentially expressed mRNAs was validated via RT-qPCR, meaning that many expression changes detected by sequencing remain to be independently confirmed. Third, the sample size was relatively small, which may have reduced the inferential strength and external validity of the results. Fourth, the study was limited to an animal model, and the findings may not fully translate to human physiology. Fifth, our CIH mouse model was not quantitatively calibrated against polysomnography-derived clinical indices, such as the apnea–hypopnea index (AHI) or oxygen desaturation index (ODI). Sixth, we did not longitudinally monitor body weight trajectories or general-condition indices such as food intake and stress during CIH exposure. Because CIH can affect feeding behavior and stress responses, particularly in obese ob/ob mice, unmeasured changes in these factors may have contributed to inter-individual variability and could partially confound interpretation of the observed metabolic and pancreatic outcomes. Seventh, we did not perform quantitative histological scoring or marker-based immunostaining/functional assays to evaluate pancreatic endocrine/exocrine function and injury-related pathways (e.g., inflammation, apoptosis, ER stress, and oxidative stress), because additional tissue/sections were not reserved in the original experimental design. Future studies will incorporate systematic morphometric quantification, targeted biomarker assessments, and functional readouts. Despite these limitations, the present study offers important insights into the transcriptomic landscape of pancreatic tissue injury under intermittent hypoxia and lays a foundation for future mechanistic and translational research.

## 5 Conclusion

In summary, this study demonstrates that mRNAs are differentially expressed in an OSA-induced pancreatic injury model using leptin-deficient ob/ob mice, suggesting their potential involvement in the pathogenesis of CIH-related pancreatic dysfunction. These findings highlight the therapeutic potential of targeting mRNA to ameliorate OSA-associated diabetes and further support the feasibility of mRNA-based interventions in this context. Nevertheless, additional studies are required to delineate the specific functions and mechanistic basis of mRNAs in CIH-induced pancreatic injury.

## Data Availability

The raw data supporting the conclusions of this article will be provided by the authors.
