# Supplementary material for: mRNA expression profiling and pathway analysis of chronic intermittent hypoxia–induced pancreatic injury in ob/ob mice
Source: Front Physiol. 2026 Feb 25;17:1740223. doi: 10.3389/fphys.2026.1740223 (PMC12975469; doi:10.3389/fphys.2026.1740223)
Supplement: Supplementary file 2 [file DataSheet1.pdf]

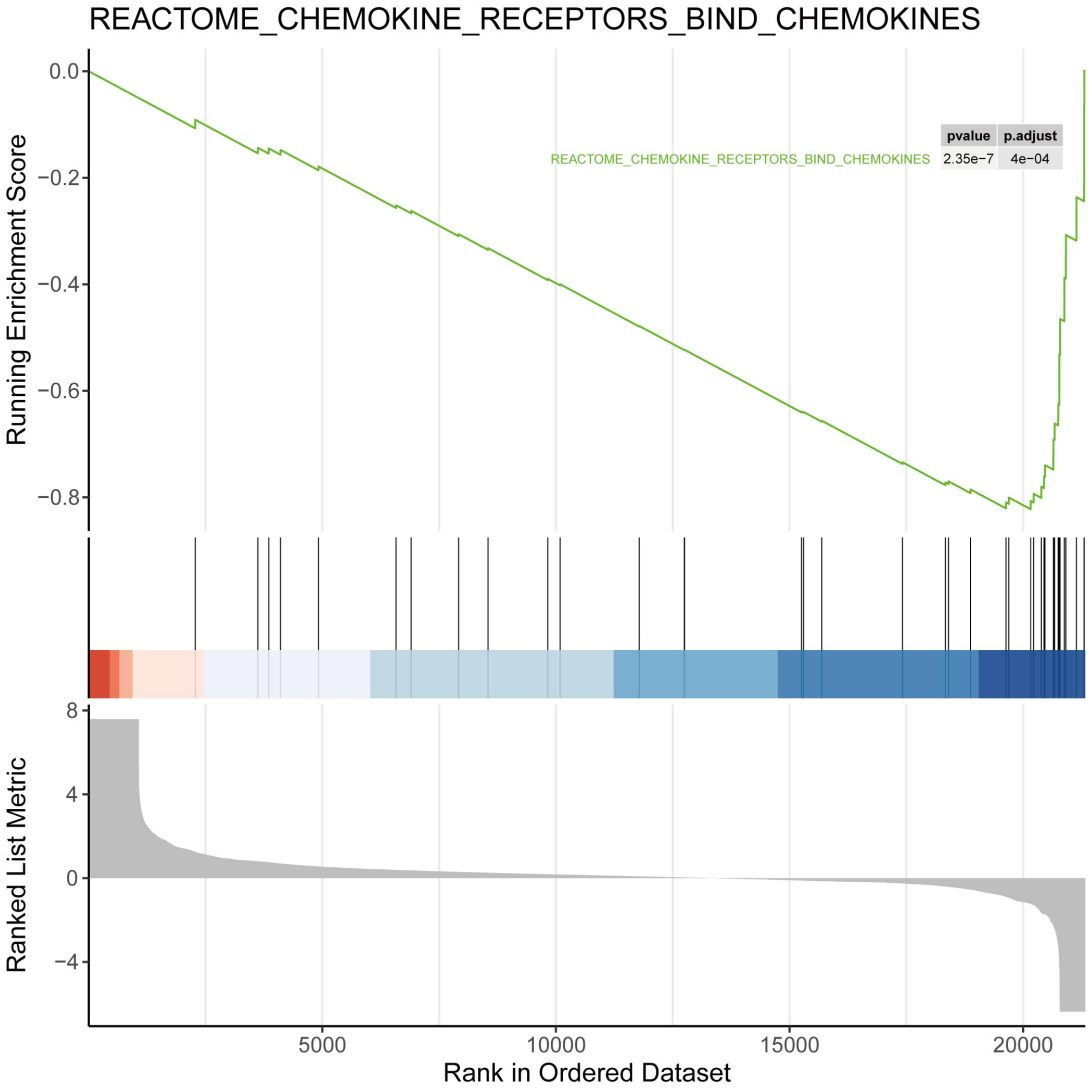

Supplementary Figure S1. Rank-based GSEA enrichment plot for chemokine receptors bind chemokines.
